# Antidepressants: Relationship to the Time to Psychiatric Readmission and Probability of Being in Hospital in Depressive Patients

**DOI:** 10.3389/fpubh.2014.00040

**Published:** 2014-05-08

**Authors:** Ingeborg Warnke, Carlos Nordt, Jörn Moock, Wolfram Kawohl, Wulf Rössler

**Affiliations:** ^1^Competence Tandem, Innovation Incubator, Leuphana University Lüneburg, Lüneburg, Germany; ^2^Department of Psychiatry, Psychotherapy and Psychosomatics, Center for Social Psychiatry, University Hospital of Psychiatry, Zurich, Switzerland; ^3^University of Zurich, Zurich, Switzerland; ^4^Laboratory of Neuroscience, LIM27, Faculty of Medicine, University of Sao Paulo, Sao Paulo, Brazil

**Keywords:** antidepressants, sertraline, depression, time to psychiatric readmission, probability of being in hospital, time hazard models

## Abstract

**Introduction:** Although antidepressants play a major role in the treatment of patients with depression, it is unclear which specific antidepressants are more efficacious than others. This study aims to analyze the relationship between several antidepressant substances and the time to readmission as well as the probability of being in hospital in a given week by using prescription data.

**Methods:** The database was health-insurance claim data from the new Federal States in Germany. The analysis consisted of all patients with unipolar depression at their index admission in 2007 (*N* = 1803). Patients were followed up for 2 years after discharge from index hospitalization. Statistical analyses were conducted by discrete-time hazards models and general estimation equation models, accounting for various predictors.

**Results:** Of all prescribed antidepressant substances, sertraline was related to an increased time to readmission by 37% and to a reduction in the probability of being in hospital in a given week by 40%. However, it was prescribed to only about 5% of the patients.

**Conclusion:** In this study, only sertraline appeared to have clinical and economic advantages. It is remarkable that just a minority of patients received sertraline in our study, thus differing from the prescription pattern in the US.

## Introduction

Besides psychotherapy and cognitive behavioral therapy, antidepressant medication is a common treatment in patients with depression (1, 2) and recommended in clinical guidelines (3, 4). The treatment with antidepressants is reported to be effective when compared to placebos, at least in patients with acute and moderate to severe depression (5). First generation antidepressants [tricyclic antidepressants (TCAs) and monoamine oxidase inhibitors (MAOIs)] were the mainstays for pharmacological treatment of depressive disorders for many years (6, 7). In the past decades, several new drugs (second-generation antidepressants) with improvements in safety and tolerability have been introduced (7–9). They include selective serotonin reuptake inhibitors (SSRIs; e.g., escitalopram, sertraline) and serotonin–norepinephrine reuptake inhibitors (SNRIs; e.g., venlafaxine, duloxetine). In the US and Europe, SSRIs are most frequently prescribed (1, 9, 10). However, there are less prescriptions of SSRIs in Germany compared to other European countries (10). German studies also suggest a preference for other antidepressants according to the German anatomical therapeutical chemical code (ATC-index) (e.g., venlafaxine, mirtazapine) (11, 12) or for TCAs (e.g., trimipramine) (13).

However, despite the general efficacy of antidepressants, up to now it is not clear which single antidepressant drug is the obvious first-line treatment of depression (9). Recent reviews have stated that sertraline might be superior to its competitors with respect to efficacy, acceptability, and tolerability (6, 14, 15) as well as acquisition cost (15). Other studies argue in favor of escitalopram (16), venlafaxine (17), or mirtazapine (18) or did not find any difference between second-generation antidepressants with respect to efficacy (19–22).

Previous studies on antidepressants and outcome have mostly used experimental trials in terms of head-to-head comparisons (6, 22) or placebo-controlled studies (21, 22). However, naturalistic studies allow for a higher ecological validity, fewer ethical concerns, and less restriction on sample size as well as on observation period. To our knowledge, evidence is rare particularly with respect to antidepressants and the long-term outcome measure “time to readmission” (23, 24). Such studies give an insight into the prevention of early readmission by appropriate treatment in clinical practice. The time to readmission serves as a quality indicator that might be lengthened by attending to stability of clinical condition (25). To date, the most valid determinant of psychiatric readmission is the number of previous admissions (26).

Regarding the economical perspective, it is important to know more about the relationship between antidepressants prescribed in outpatient care and the probability of being in hospital in a given time period. In general, findings on the cost-effectiveness of antidepressants are mixed (1). The main cost component in depressive patients is inpatient care. In a recent German study, inpatient care comprised 43.9% of the total annual direct costs of patients with depression (27).

The aim of this study was to analyze the relationship between first- and second-generation antidepressants in outpatient care with respect to (a) the time until readmission and (b) the status of “being in hospital at a given time period” in depressive patients. We analyzed prescription data reflecting treatment under ecological conditions and we controlled for several variables.

## Materials and Methods

### Study design and data source

This study is a retrospective analysis using electronic health-insurance claims data from a German statutory health-insurance company, located in the new Federal States (AOK Plus). The AOK Plus covers about three million insured persons; the majority of clients live in Saxony and Thuringia. We selected relevant information out of several databases on depressive patients [a depressive episode (F32, ICD-10) or a recurrent depressive disorder (F33, ICD-10)]. The time span ranged from January 1, 2007 to September 30, 2010. Hospital data consisted of *N* = 71,490 inpatient episodes due to a mental disorder (F-diagnosis by ICD-10) treated in a psychiatric or psychosomatic unit. Further, we considered data on prescribed drugs, diagnoses, episode-specific variables, and personal characteristics (age, sex, residential region, type of insurance, and period of coverage).

### Sample selection

**Figure 1:**
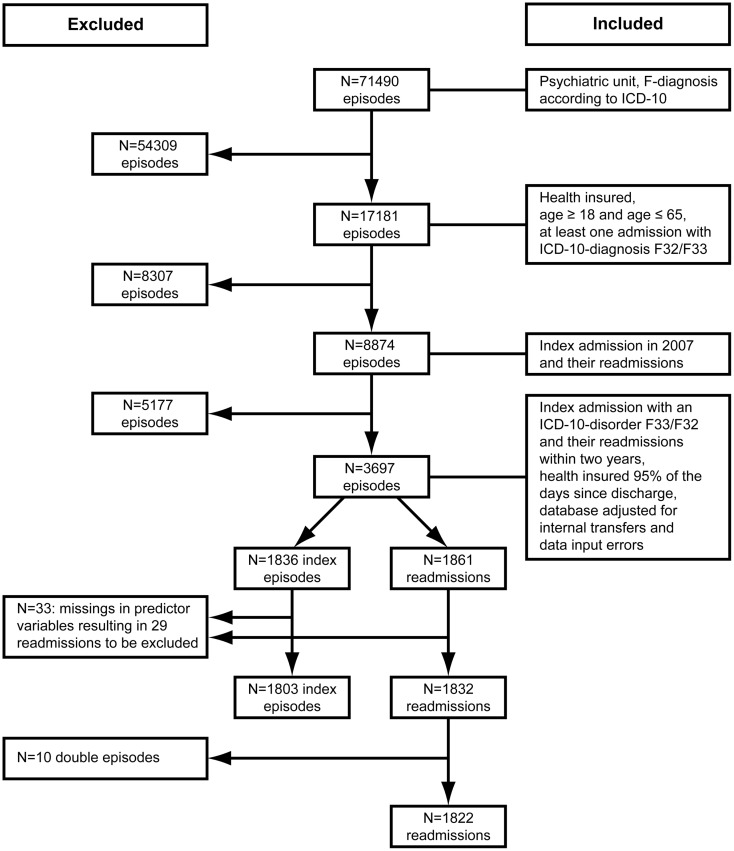
**Sample selection process referring to inpatient episodes**.

Figure 1 shows the process of sample reduction by specific selection criteria according to the research issue of our study: (a) patients between 18 and 65 years; (b) at least one inpatient episode with a disorder F32/F33; (c) at least one hospital admission in the year 2007; (d) health-insured at least 95% of the time within the observation period, referring to the health care system in Germany; (e) adjusting database with respect to internal transfers and data input errors; and (f) complete data in all predictor variables.

Steps (b) and (c) were conducted to select the index episode for depressive patients. The observation period started with the discharge from the index episode. Irrespective of the F-diagnosis in the follow-up, we considered all readmissions of those patients primarily diagnosed with a depressive disorder. Under clinical considerations, we assumed that the basic symptomatology might persist over 2 years and that, due to clinical comorbidity, diagnoses might change over subsequent inpatient episodes.

Step (d) served to include only those patients who were health-insured most of the time within the observation period of 2 years. The aim was to obtain a sample that was comparable in terms of the probability of returning to hospital. In Germany, not being insured might be due to job transitions, lack of registration at the employment office, or to changes of insurance company.

Step (e) was taken to improve data quality of the sample fulfilling the inclusion criteria. We only considered data with complete information on predictor variables. Of *N* = 1836 index episodes, severity of illness was missing in 30 patients with unspecified disorder (F32.8/F32.9; F33.8/F33.9). Employment status at index admission was missing in three patients. The final sample consisted of *N* = 1803 patients who had in total *N* = 1832 readmissions. Ten patients experienced more than one readmission within the same week. Accordingly, we only considered the last admission, which finally resulted in *N* = 1822 readmissions.

### Predictor variables

Sociodemographic variables were gender (female vs. male), age (in years), and employment status (employed vs. unemployed) in the quarter of index admission.

Illness-related variables at the index episode were main diagnosis F32 vs. F33 (yes vs. no), severity of illness (mild to moderate vs. severe), secondary diagnosis according to ICD-10 ((F0-F6; A0-E9, G0-Z9), yes vs. no). For the final statistical models, we only considered secondary disorders prevalent in about 5% (at least 4.5%) of the sample at the index episode (week = 0): at least one substance use disorder, at least one anxiety disorder, at least one personality disorder, at least one somatic/neurological disorder.

#### Time-varying predictor variables

##### Medication

We considered every prescription in outpatient care of several types of drugs at weekly time intervals. Finally, we only selected drugs that were taken by about 5% (at least 4.5%) of the sample at the start of the observation period. The data finally consisted of three different types of antidepressant according to the ATC-index (11): non-selective monoamine-reuptake inhibitor (NSMRI), SSRI, and others (not attributable to one of the previous groups). Further, we examined the following substances: citalopram (SSRI), sertraline (SSRI), escitalopram (SSRI), trimipramine (NSMRI), mirtazapine (others), venlafaxine (others), and duloxetine (others).

Other drugs contained sedatives, psychotropic drugs other than antidepressants (mostly antipsychotics), and drugs for all kinds of somatic health problems. The number of defined daily doses (DDD) (11) per medication package served as an estimate for the duration of medication use.

With every new hospitalization episode, we used the corresponding type of admission (emergency vs. regular, time-variant), number of episode (logarithmized), length of previous admission in days (logarithmized) as predictors for the duration until readmission. For the latter variables, we used the natural logarithm.

### Statistical analysis

For each individual, we constructed a person–period dataset of 105 weekly time intervals, where we could easily set the time-dependent predictor variables (28). We implemented two outcome variables: (1) hospital admission yes (event = 1) vs. no (event = 0), (2) status of hospital stay (in hospital, yes, event = 1 vs. out of hospital, no, event = 0).

To analyze the first outcome “time to readmission,” we applied extended survival models that are able to analyze more than one event within the observation period. Therefore, the time indicator was reset to zero after each hospitalization episode (29). Thus, patients were assumed not to be at risk for a subsequent event until the current event had terminated. As a result, we observed a minimum of 47 time intervals and a maximum of 105 time intervals per patient. The person–period dataset for the first outcome finally consisted of *N* = 181,049 observations and *N* = 1822 events.

To analyze the second outcome “being in hospital that week” we considered all 105 time intervals per subject, thus the person–period dataset for the second outcome was larger consisting of *N* = 189,315 observations and *N* = 10,088 events.

For the time to readmission, we applied discrete-time hazard models (28) allowing for a parsimonious representation of the variable “time.” Thus, the models fit the shape of the logit-hazard profile. The hazard probability describes the proportion of the risk set that experiences the event during that period. To analyze the probability of being in hospital, we applied a general estimation equation (GEE) with binomial response for correlated data. We used the natural logarithm (log) of time for all analyses.

We were interested in the question if antidepressants have an additional contribution to sociodemographic, clinical, and further medication variables on the outcome variables of interest. Accordingly, we conducted three models for each outcome. First, we used time and episode as predictors for the time to readmission, and only time variables for the probability of being in hospital. Concerning the latter, the number of previous inpatient episodes was not relevant. These models provide a standard against which later models (including further predictors) can be compared (30). Second, we added sociodemographic and clinical predictor variables and finally all drug variables.

For the analyses, we used the genlin-procedure implemented in SPSS 18. An independent working correlation matrix was estimated by a subject variable (29) to account for multiple episodes and their probable correlation within subjects. We used all medication variables irrespective of the significance level. With respect to the other probable predictors, the level of significance was set to <0.01 in order to consider only the most important ones.

## Results

### Descriptive results

Of the *N* = 1803 patients, *N* = 779 (43%) had at least one readmission over the study period of 2 years. The maximum number of readmissions per patient during the follow-up period was 22.

Table 1 summarizes the sample characteristics. The sample consisted of almost twice as many female as male patients. The median age was 48 years. Patients had a median length of stay of 34 days with upper and lower quartiles of 53–18 days [=interquartile range (IQR)]. Most of the patients had a depressive episode (F32). About 50% of the patients were considered to be severely ill. The most frequent secondary disorders were somatic/neurological disorders as well as anxiety, personality, and substance use disorders. Regular admissions were more frequent than emergency admissions. About half of the patients (*N* = 934, 51.8%) received antidepressant medication when considering only the substances relevant for this study (see above). Most patients used antidepressants other than SSRIs or NSMRIs, especially the substances mirtazapine (16.5%) and venlafaxine (16.3%). Only a minority received sertraline (4.8%). Less than 10% used sedatives, whereas more than one-third used other psychotropic drugs and almost half of the patients used somatic medication.

**Table 1:** **Sample characteristics at time after discharge from index hospitalization**.

Characteristic		Total, *N* (%)
Sociodemography	Gender, female	1149 (63.7)
	Age	48 (55–40)
	Employment, yes	643 (35.7)
Clinical variables, secondary disorders	Recurrent depressive disorder (F33) [vs. depressive episode (F32)]	792 (43.9)
	Severity of disorder, severe (vs. light to moderate)	908 (50.4)
	Organic disorder	38 (2.1)
	Substance use disorder	346 (19.2)
	Psychotic disorder	48 (2.7)
	Affective disorder	7 (0.4)
	Anxiety disorder	491 (27.2)
	Behavioral disorder	66 (3.7)
	Personality disorder	371 (20.6)
Admission variables	Length of stay	34 (53–18)
	Type of admission, emergency	481 (26.7)
Medication, substances	NSMRI	106 (5.9)
	SSRI^a^	350 (19.4)
	Other	625 (34.7)
	Sedatives	138 (7.7)
	Psychotropics, others	632 (35.1)
	Somatic medication	773 (42.9)
	Citalopram/escitalopram (SSRI)	272 (15.1)
	Sertraline (SSRI)	86 (4.8)
	Trimipramine (NSMRI)	106 (5.9)
	Mirtazapine (others)	297 (16.5)
	Venlafaxine (others)	293 (16.3)
	Duloxetine (others)	106 (5.9)

*^a^*N* = 8 patients used two different SSRI drugs at the same time (resulting in *N* = 350 patients and *N* = 358 prescriptions)*.

### Discrete-time hazards model: predictors of time to readmission

As described above, we computed three statistical models when analyzing the time to readmission by consecutively adding predictor variables (Table 2). First, we only considered the variable time and number of episodes (readmissions). Second, we added patient variables, mostly related to the index episode. Third, we also examined several drug variables.

**Table 2:** **Discrete-time hazards model: predictors of time to readmission**.

Characteristic	Model 1		Model 2		Model 3	
	OR	95%-CI	OR	95%-CI	OR	95%-CI
Constant	0.04	0.04–0.05	0.05	0.04–0.06	0.05	0.04–0.07
Time (log)	0.53	0.51–0.56	0.52	0.50–0.55	0.51	0.48–0.54
Number of episodes (log)	2.31	2.14–2.50	1.95	1.78–2.13	1.91	1.74–2.10
**Sociodemography**						
Age	–	–	1.00	0.99–1.01	1.00	1.00–1.01
Gender, female	–	–	1.13	1.02–1.25	1.11	0.99–1.23
Employment, yes	–	–	0.76	0.67–0.85	0.77	0.68–0.86
Clinical variables at index						
Secondary disorder, personality	–	–	0.87	0.67–1.09	0.84	0.66–1.08
Secondary disorder, personality × time (log)			1.15	1.05–1.26	1.16	1.06–1.28
Secondary disorder, substance abuse	–	–	1.40	1.25–1.57	1.39	1.23–1.57
Severity of disorder at index	–	–	1.16	1.04–1.30	1.15	1.03–1.28
Main diagnosis, F33	–	–	1.20	1.08–1.35	1.20	1.07–1.34
**Admission variables^a^**						
LOS (log)	–	–	0.87	0.84–0.91	0.87	0.83–0.91
**Medication^a^**						
Sedatives	–	–	–	–	0.94	0.66–1.35
Sedatives × time (log)	–	–	–	–	1.32	1.16–1.50
Psychotropics, others	–	–	–	–	1.04	0.93–1.16
Somatic medication	–	–	–	–	0.92	0.82–1.03
**Substances^a^**						
Citalopram/escitalopram (SSRI)	–	–	–	–	1.09	0.94–1.27
Sertraline (SSRI)	–	–	–	–	0.63	0.49–0.81
Trimipramine (NSMRI)	–	–	–	–	1.06	0.86–1.32
Mirtazapine (others)	–	–	–	–	0.96	0.84–1.10
Venlafaxine (others)	–	–	–	–	1.01	0.87–1.17
Duloxetine (others)	–	–	–	–	0.92	0.77–1.10

*In total, we considered *N* = 181,049 observations (person–period dataset) with *N* = 1822 events*.

*^a^ Time-varying covariates*.

*Log = natural logarithm*.

In all three models, the risk of readmission decreased over time. The time to readmission was reduced with the number of episodes. Models 2 and 3 show that the time to readmission was reduced for the following clinical parameters: recurrent depressive disorder at the index episode, substance use disorder and more severe illness. Moreover, we found an interaction effect between personality disorder and time: although patients with a personality disorder initially had a longer time to readmission, the time was reduced by half over time. Employment at the index episode and longer hospital stays lengthened the time to readmission. Regarding medication variables in model 3, sertraline (SSRI) was the only significant antidepressant reducing the risk of readmission by an odds ratio (OR) of 0.63 (95% CI = 0.49–0.81) meaning a lengthening of the time to readmission by 37% (95% CI = 19–51%). Further, we found an interaction effect between sedatives and time: the prescription of sedatives shortened the time to readmission within 2 years after index hospitalization. We did not find an indication that sertraline had a different effect with respect to severity of the disorder (results not shown).

### GEE model: predictors of being in hospital in a given week

According to the procedure described above, we again computed three statistical models by analyzing probable predictors of the variable “hospital stay in a given week” (yes: event = 1 vs. no event = 0) (Table 3). Regarding models 2 and 3, having a recurrent depressive disorder at index episode, having a secondary personality or substance disorder, and being more severely ill at the index episode increased the probability of being in hospital. Employment status decreased the risk of being in hospital. With respect to drug variables, using somatic medication or using sertraline (SSRI) both reduced the weeks of being in hospital by 40% (95% CI = 32–48%).

**Table 3:** **GEE model: predictors of being in hospital in a given week**.

Characteristic	Model 1		Model 2		Model 3	
	OR	95%-CI	OR	95%-CI	OR	95%-CI
Constant	0.06	0.04–0.07	0.03	0.02–0.04	0.02	0.02–0.04
Time (log)	2.24	1.53–3.27	2.26	1.54–3.31	2.30	1.56–3.39
Time (log) × time (log)	0.72	0.59–0.86	0.71	0.59–0.86	0.71	0.59–0.86
Time (log) × time (log) × time (log)	1.03	1.01–1.06	1.03	1.01–1.06	1.03	1.01–1.06
Sociodemography						
Age	–	–	1.01	1.00–1.01	1.01	1.01–1.02
Gender, female	–	–	1.22	1.02–1.46	1.27	1.07–1.52
Employment, yes	–	–	0.68	0.56–0.83	0.65	0.54–0.79
Clinical variables at index						
Secondary disorder, personality	–	–	1.36	1.11–1.66	1.38	1.13–1.68
Secondary disorder, substance abuse	–	–	1.52	1.25–1.85	1.52	1.26–1.85
Severity of disorder at index	–	–	1.29	1.09–1.54	1.29	1.09–1.53
Main diagnosis, F33	–	–	1.32	1.11–1.58	1.38	1.16–1.64
Medication^a^						
Sedatives	–	–	–	–	1.10	0.91–1.34
Psychotropics, others	–	–	–	–	0.92	0.81–1.04
Somatic medication	–	–	–	–	0.60	0.52–0.68
Substances^a^						
Citalopram/escitalopram (SSRI)	–	–	–	–	0.97	0.78–1.20
Sertraline (SSRI)	–	–	–	–	0.60	0.42–0.86
Trimipramine (NSMRI)	–	–	–	–	0.81	0.60–1.09
Mirtazapine (others)	–	–	–	–	0.91	0.76–1.09
Venlafaxine (others)	–	–	–	–	0.90	0.74–1.09
Duloxetine (others)	–	–	–	–	0.92	0.72–1.18

*We considered *N* = 189,315 observations (person–period dataset) with *N* = 10,088 events*.

*^a^ Time-varying covariates*.

*Log = natural logarithm*.

Again, the interaction between sertraline and severity of the disorder was not significant (results not shown).

## Discussion

This study aimed to analyze the relationship between antidepressant substances in outpatient care and time to readmission or probability of being in hospital by prescription data. We were interested in treatment under ecological conditions with the respective clinical and economic implications.

In our study, the antidepressant sertraline was linked with a lengthened time to readmission and a reduced probability of being in hospital. Our findings were irrespective of the severity of the disorder at the index episode (see above). Due to differences in design and time span, it is difficult to compare our results with experimental trials. As stated, previous evidence on antidepressants is contradictory. The observation period of experimental trials mainly ranges between 6 and up to 24 weeks. However, interestingly, recent reviews on acute-phase treatment particularly favored sertraline over several new antidepressants in terms of efficacy, acceptability, and tolerability (14, 15). Another interesting placebo-controlled study showed that the time to relapse was significantly longer for depressive patients receiving sertraline compared to a placebo (31). Further, continuation sertraline treatment was associated with improved quality of life.

Due to lack of data, it was not possible to account for certain variables in our study. For example, it remains unclear if patient adherence has been particularly high in patients using sertraline. Further, physician preferences, dosage, toxic effects, discontinuation symptoms, pattern of medication use, treatment latency, social functioning, or quality of life could not be explored.

Only 5% of the patients received sertraline in this study. Most patients used venlafaxine or mirtazapine. In Germany, sertraline and venlafaxine belong to the group of antidepressants licensed for recurrence prophylaxis in unipolar depression (4). The prescription pattern in our study is contrary to European data (10) and studies conducted in the US (1, 2, 30, 32), reporting that SSRIs were most frequently prescribed. Further, at least in the US, sertraline is the most prescribed single agent (1). It remains unclear why sertraline was so rarely prescribed in our sample. One explanation might be undertreatment (see below). Further, the use of SSRIs might imply health-related disadvantages, even if sertraline appears to be well tolerated. Common side effects of SSRIs are sexual dysfunction in men, CNS, and anticholinergic effects as well as gastrointestinal distress (3). The association between SSRIs and cancer or suicidality has yet to be proven (33–35).

As mentioned above, antidepressants other than sertraline were not significantly related to our outcome variables. Accordingly, the usefulness of antidepressants could be viewed critically, as argued by other authors (36). Further, about 50% of the patients in our study did not receive any antidepressant relevant for our study in the week after discharge from index hospitalization. This corresponds to findings on depressive outpatients with up to more than half not receiving depression-specific treatment (37–39). Those numbers of patients with antidepressant treatment are unexpectedly low, at least when compared to studies on depressive inpatients (12). The role of factors such as utilization of prescription-free herbal antidepressants not registered in our prescription database or non-compliance to prescribed antidepressants remains unclear. At least the first is unlikely because patients without prescribed antidepressants did not differ from patients with antidepressants with respect to severity of illness (results not shown). Further, lack of data quality could have played a role: lack of information on dispersion of antidepressant medication during inpatient treatment might imply that some patients continued using medication delivered to them in the hospital (see limitations below). When considering several antidepressant substances not included in our study due to their small prevalence (≈ <5%), the number of patients using antidepressant medication at baseline increased to only 58%. Besides sertraline, sedatives and somatic medication were significant in our study. Sedatives, used by about 8% of the patients, appeared to increasingly reduce the time to readmission over the study period (Table 2). The use of sedatives seems to be more pronounced in patients with readmissions and is probably related to the acute-phase of mental illness. Possibly, misuse or dependency played a role. The German guidelines recommend sedatives as a short-term treatment to overcome the treatment latency of the antidepressant effect and acute agitation (4).

Concerning somatic medication, we found a reduction of the risk of being in hospital. The use of somatic medication might serve as an indicator for the predominance of somatic compared to psychiatric symptoms, probably also affecting treatment-seeking behavior.

We controlled for time and the episode-specific variables as number of previous inpatient episodes and length of stay. Our finding of a decreasing risk of readmission over time after index hospitalization corresponds with another study on schizophrenic patients (40). In line with previous studies (24, 26, 41), we have found that a higher number of previous psychiatric admissions reduces the time to readmission. This might be related to a treatment-seeking behavior (26) and/or the vulnerability to further crisis and hospitalizations (41). Regarding length of stay, shorter previous hospital stay was associated with a reduced time to readmission. It remains unclear whether this refers to the revolving-door phenomenon and if patients were previously discharged too early. In a study on unipolar depression, a first hospitalization of between 15 and 30 days was associated with the shortest mean cumulative length of stay (42).

Further relevant predictor variables in our study were employment status, severity of illness, diagnosis, and secondary diagnosis. Employment at index hospitalization had a preventive effect on the outcomes. Accordingly, previous studies showed a link between employment and a lower risk of hospital readmission (43–45). In particular, employed mentally ill patients seem to prefer psychiatric outpatient compared to inpatient treatment (45), probably to ensure keeping their job. Illness-related variables were associated with a shorter time to readmission and a higher probability of being in hospital. Previous studies on time to readmission correspond with these results (24). In general, studies on psychiatric inpatient length of stay suggest that treatment and organizational variables have a higher impact than patient variables (46).

The limitations of this study are the following: our study was not an experimental trial, which is a strength and a weakness at the same time. On the one hand, our data have higher ecological validity considering that the choice of pharmacotherapy is driven primarily by patient choice (9). On the other, only experimental trials allow for direct causal conclusions (47). In our dataset, important variables were missing (see above), e.g., medication prescription during hospitalization, compliance, toxicity of the antidepressant drugs, or quality of life. An examination of the latter outcome criteria could give further insight into the value or utility of the respective antidepressants. Concerning medication prescription during hospitalization, the same drugs were equally distributed during the time in and out of hospital (results not shown). Data like illness severity or employment were considered only for the time of the index admission. This was because we were interested in baseline patient characteristics. The validity of the dataset remains unclear, but we aimed to improve data quality by sample selection. Further, we compared patients with different severity levels and treatment histories. However, for this analysis we were interested in all depressive patients. A reanalysis of our research question with a larger sample and a higher number of patients receiving sertraline might be promising.

In summary, this study analyzed antidepressants as possible predictors of the time to readmission and the probability of being in hospital in a given time period. Sertraline was the only substance with a preventive effect concerning time to readmission and probability of being in hospital. Its prescription might have positive clinical and economic consequences. However, this substance was prescribed only to a minority of patients, which is contrary to the prescribing pattern in the US. Future research on further outcome parameters like toxic effects of antidepressants and quality of life is necessary to comprehensively assess the utility of antidepressant drugs.

## Author Contributions

Ingeborg Warnke designed the study, cleaned and analyzed the data, and drafted and revised the paper. Carlos Nordt wrote the statistical analysis plan and revised the draft paper. Wulf Rössler, Jörn Moock, and Wolfram Kawohl designed the study and revised the draft paper.

## Conflict of Interest Statement

Wolfram Kawohl received compensation as a consultant for Janssen-Cilag; Wolfram Kawohl received compensation for scientific talks from Bristol-Myers Squibb, Eli Lilly, Essex, Janssen-Cilag, and Vifor. Wolfram Kawohl received sponsoring for travel costs and participation in scientific meetings from Astra Zeneca, Bristol-Myers Squibb, Lundbeck, Janssen-Cilag, and Vifor. Wolfram Kawohl received funding for investigator-initiated projects from Eli Lilly and Bristol-Myers Squibb. All other authors declare that they have no conflicts of interest to report linked to this manuscript. The Review Editor Leandro Da Costa Lane Valiengo declares that, despite being affiliated to the same institution as author Wulf Rössler, the review process was handled objectively and no conflict of interest exists.
